# Mathematical Models of HIV-1 Dynamics, Transcription, and Latency

**DOI:** 10.3390/v15102119

**Published:** 2023-10-19

**Authors:** Iván D’Orso, Christian V. Forst

**Affiliations:** 1 Department of Microbiology, University of Texas Southwestern Medical Center, Dallas, TX 75390, USA; ivan.dorso@utsouthwestern.edu; 2 Department of Genetics and Genomic Sciences, Department of Microbiology, Icahn School of Medicine at Mount Sinai, New York, NY 10029, USA

**Keywords:** HIV-1, transcription, latency, reactivation, stochastic fluctuations, mathematical modeling

## Abstract

HIV-1 latency is a major barrier to curing infections with antiretroviral therapy and, consequently, to eliminating the disease globally. The establishment, maintenance, and potential clearance of latent infection are complex dynamic processes and can be best described with the help of mathematical models followed by experimental validation. Here, we review the use of viral dynamics models for HIV-1, with a focus on applications to the latent reservoir. Such models have been used to explain the multi-phasic decay of viral load during antiretroviral therapy, the early seeding of the latent reservoir during acute infection and the limited inflow during treatment, the dynamics of viral blips, and the phenomenon of post-treatment control. Finally, we discuss that mathematical models have been used to predict the efficacy of potential HIV-1 cure strategies, such as latency-reversing agents, early treatment initiation, or gene therapies, and to provide guidance for designing trials of these novel interventions.

## 1. Introduction

Viral latency refers to the period during which the human immunodeficiency virus (HIV-1) remains dormant and hidden in certain immune cells of the body, primarily CD4 T cells, myeloids, and microglia [1,2,3,4,5,6,7]. This latent phase of infection poses a significant challenge in curing HIV-1 and eliminating the disease globally. Antiretroviral therapy (ART) is highly effective in suppressing viral replication and reducing the viral load in an infected individual. However, it is unable to completely eliminate the virus due to the presence of the latent reservoir. To gain a better understanding of the establishment, maintenance, and potential clearance of latent HIV-1 infection, researchers have turned to mathematical models. These models provide a framework for simulating and studying the complex dynamics of viral replication, viral decay, and the interaction between the virus and the immune system. Viral dynamics models have been instrumental in explaining various phenomena observed in HIV-1 infection and treatment. For instance, these models: (1) have helped explain the multi-phasic decay of viral load observed during ART [8,9,10]; (2) have shown that the initial rapid decline in viral load is followed by a slower decay phase, in part contributed by the persistence of the latent reservoir [11] (other factors are the decay of defective provirus inducing an even slower delay or extensive proliferation of infecting cells counteracting a slower decay [12]); (3) have been used to evaluate the benefits of early treatment initiation and gene therapies in cure research [13]; (4) have helped assess the impact of early ART initiation on reducing the size of the latent reservoir and the likelihood of achieving a functional cure [14]; and (5) have aided in designing clinical trials for novel interventions by estimating sample sizes, treatment duration, and outcome measures required to assess efficacy [15,16]. Mathematical models have also shed light on the early seeding of the latent reservoir during acute HIV-1 infection and the limited inflow of cells into the reservoir during treatment. These models have revealed that the establishment of latency occurs rapidly after initial infection and that ongoing low-level viral replication contributes to the replenishment of the reservoir over time. Additionally, viral dynamics models have helped explain the phenomenon of viral blips, which are transient increases in viral load during ART [13,17]. These blips can occur due to various factors, such as low-level viral replication, spontaneous activation of latently infected cells, or fluctuations in immune control. Moreover, mathematical modeling has played a crucial role in predicting the efficacy of potential HIV-1 cure strategies. For example, models have been used to assess the impact of latency-reversing agents (LRAs), which aim to reactivate latent HIV-1, making it vulnerable to clearance by the immune system or due to cytopathic effects. By simulating the effects of different LRAs and treatment combinations, models can provide valuable predictions on the potential success of these strategies.

In summary, mathematical models have significantly contributed to our understanding of HIV-1 latency and its implications for therapeutic strategies [18]. By simulating the complex dynamics of viral replication, latency, and immune responses; these models have provided insights into the role of the persistent latent HIV-1 reservoir and have guided the development and evaluation of potential cure interventions.

## 2. History of Mathematical Models of HIV-1

In the late 1980s, shortly after the discovery of HIV-1, the first models for viral infection within individuals were developed. These models were influenced by the existing mathematical epidemiology models, particularly the compartmentalized models like the Susceptible → Infectious → Susceptible models (SIS) and Susceptible → Infectious → Resistant (SIR) models introduced by Kendrick and McCormack in the early 1900s, which are used widely to describe the spread of infections between individuals in a population [19,20,21]. The SIS model assumes that individuals can become infected and then recover, but without developing long-term immunity, so they can become susceptible again. On the other hand, the SIR model considers that individuals who recover from infection acquire long-term immunity and assumed to become resistant. However, when it came to describing the dynamics of the virus within an individual’s body, a different type of model called “viral dynamics” models was developed. This model aimed to capture the spread of the virus between infected cells within the body of a single individual [22]. They focused on factors such as the replication and clearance rates of the virus, as well as the immune response of the individual. Since the development of these viral dynamics models for HIV-1, similar modeling approaches have also been applied to understand the dynamics of other human viral infections. Examples include Hepatitis B [23,24,25] and C [26,27], influenza [28], dengue [29,30], and herpes simplex virus [31], but will not be discussed in this Review.

**Figure 1:**
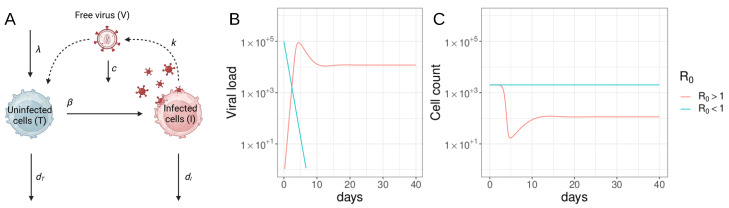
The basic viral dynamics model. (**A**) Cell population processes that are simulated in the mathematical model (Equation (1)). (**B**,**C**) Example trajectory of viral load (V) and uninfected target cells (CD4 T cells, T) when $R_{0}>1$ (red) and $R_{0}<1$ (blue). Graphs were generated by numerically integrating Equation (1) with parameters λ=100 cells/µL, $\beta =7\times 10^{-5}$ or 0/day/(virus/mL), k=150 virus/cell, $d_{T}=0.05/$day, $d_{I}=0.7/$day, c=1.7/day using JSim v2.21 with standard integration parameters [32]. (Figure adapted from Hill [18] and created by part (**A**) with BioRender.com).

The basic version of the viral dynamics model for HIV-1, described in Section 2.1.1, Equation (1), and Figure 1, has been instrumental in gaining a deep understanding of HIV-1 dynamics. Despite its simplicity, this model has provided valuable insights by accurately predicting the time-course of viral load during acute HIV-1 infection.

According to the model, the viral load exhibits an initial exponential increase, followed by a peak, and then a decline to a steady state (as shown in Figure 1B). Notably, the model revealed that the post-peak decline in viral load could be explained even in the absence of an adaptive immune response. It demonstrated that the slower turnover rate of target cells compared to infected cells, coupled with their limited production rate, was sufficient to account for the observed drop in viral load. In this scenario, the viral population was only controlled by target cell availability. This particular dynamic regime is termed “target cell limited” [33].

As diagnostic techniques were improved, allowing for early identification of HIV-1-infected individuals and frequent monitoring, high-quality longitudinal data on viral load and CD4 T cell count became available. These data could be fitted to the viral dynamics model, enabling the estimation of parameters that govern viral dynamics [34,35].

Through these analyses, it was later determined that the post-peak decline in viral load was larger than what could be explained by target cell limitation alone. The model quantified the impact of the adaptive immune response, shedding light on its contribution to controlling viral replication and influencing the dynamics of infection challenging the initial model [33].

### 2.1. Viral Dynamics Model

#### 2.1.1. The Basic Viral Dynamics Model

The basic viral dynamics model (Figure 1) calculates the abundance of uninfected target cells of the virus (*T*), infected cells (*I*), and free virus (*V*). Target cells are generated at a rate λ, and die with rate constant $d_{T}$. Infected cells are generated by contact between target cells and free virus at a rate β, and die at rate $d_{I}$. Free virus emerge from infected cells at rate *k* and is cleared at rate *c*. The model is generally formulated as a system of ordinary differential equations (ODE)

(1) $$\begin{array}{ccc} \dot{T} & = & \lambda -\beta TV-d_{T}T\\ \dot{I} & = & \beta TV-d_{I}I\\ \dot{V} & = & kI-cV \end{array}$$

A composite quantity called the basic reproductive ratio ($R_{0}$, Equation (2)) is defined, representing the average number of secondary infected cells produced by a single infected cell over its lifetime in a population of susceptible target cells. $R_{0}$ can be calculated for this model as

(2) $$R_{0}=\frac{\lambda \beta k}{d_{T}d_{I}c}$$

The viral dynamics model used in HIV-1 research exhibits threshold behavior, similar to epidemiological models [22,36]. Mathematically, the model experiences a transcritical bifurcation. When $R_{0}$ is greater than one ($R_{0}>1$), an infection can grow and establish a chronic state. When $R_{0}$ is less than one ($R_{0}<1$), an infection will decline and eventually be cleared. In this case, the only stable equilibrium is one with no virus or infected cells (I=V=0). The transcritical bifurcation occurs at $R_{0}=1$. At this parameter, an infection would remain stationary.

Studies have estimated the range of $R_{0}$ for HIV-1 infection to be between 2 and 25 secondary infected cells, with an average of around 8 in patients [37]. This benchmark provides insight into the required efficacy of drugs to inhibit infection, where the drug efficacy (ϵ) must satisfy $\epsilon >1-\frac{1}{R_{0}}$ to achieve viral suppression within an individual. The standard formulation of the viral dynamics model (Figure 1 and Equation (1)) assumes well-mixed virus and cells throughout plasma and anatomical sites and assumes that average (deterministic) behavior is sufficient due to high levels of virus and cells. However, the model can also be simulated stochastically to account for small population sizes, chance extinction, and rare events. There are multiple stochastic formulations of the model that are equivalent to the deterministic representation but exhibit different levels of fluctuations around the average. The model typically assumes that all CD4 T cells are target cells for the virus, although some versions consider only activated cells. To more accurately describe the experimental observations and improve the predictive power, various additional details can be incorporated into the model, such as delays between infection and virion production, tracking of anti-viral immune responses, viral latency, multiple classes of infected cells, or the impact of drug treatments [22].

As described in the next section, observations of viral load trajectories during potent ART initiation have provided insights into the rate of turnover of actively infected cells. These observations first made in the mid 1990s revealed that infected cells have a rapid turnover rate, in the order of a day, despite the slow-progressing clinical nature of chronic HIV-1 infection [8,9]. This finding shifted the understanding of HIV-1 from a slow-moving infection to a dynamic one. In the early use of antiretroviral drugs as monotherapy, temporary declines in viral load were followed by resurgence to high levels (though slightly below pre-treatment levels). Models, along with genotypic testing, explained these dynamics by the appearance of drug-resistant strains and subsequent competition with wild-type strains [38,39]. Parameterized viral dynamics models demonstrated that pre-existing resistance at the start of therapy was more likely than newly emerging mutations after treatment initiation. These models, along with estimates of the mutation rate of HIV-1, suggested that at least three drugs, such as Zidovudine, Lamivudine, and Indinavir, which were tested in a randomized trial [40], would be necessary to prevent rapid failure due to drug resistance [41,42].

However, the persistence of drug resistance in HIV-1 continues to be a leading clinical problem, even against the latest generation of antiviral drugs [43]. A spectrum of models have been developed to address this problem and to describe the heterogeneous viral fitness landscape across HIV-1 patients. Shenkhar et al. [44] employed a quantum mechanical spin model that describe an expanding network of infected host derived from patient data. Their model represents the intrinsic fitness landscape of mutant viral strains, which can predict the expected diversity of the virus population. Biswas et al. [45] have been trying to understand the mutational tolerance of HIV-1 that drives the emergence of drug-resistance variance upon ART. They have developed a spin lattice model (“Potts model”) to investigate the role of epistatic interactions in the HIV-1 fitness landscape and to identify potential mutational routes of pathogen escape and drug resistance. Using their model, they confirmed an entrenchment for all three drug-target proteins: protease, reverse transcriptase, and integrase. Biswas et al. [46] were further able to show that the likelihood of resistance mutations vary widely in the patient population and between different viral sequences within a patient HIV-1 reservoir. Assessing the diversity of the entrenchment in different patient specific reservoirs may benefit future drug design strategies.

Adaptations of the basic viral dynamics model that incorporated interactions with the immune system were later developed to understand the mechanisms of HIV-1 pathogenesis and progression to the Acquired Immuno-Deficiency Syndrome (AIDS). These models aimed to explain the long period of asymptomatic infection prior to the onset of AIDS and the slow decline of CD4 T cells [47,48,49]. One early model proposed a “diversity threshold”, suggesting that continual immune escape leads to a critical level of antigenic diversity in the viral population, preventing effective control of the infection and allowing progressive immune destruction [50,51]. However, many mechanistic aspects of the HIV-1 life cycle still remain unclear, and no single model is fully supported by observations.

#### 2.1.2. Models of ART

The viral dynamics model is useful for interpreting changes in viremia when ART is initiated. In the case of fully effective drugs that prevent productive infection of bystander cells (such as entry inhibitors, reverse transcriptase inhibitors, and integrase inhibitors), treatment can be modeled approximately as β→0 in the viral dynamics model (Figure 1 and Equations (1) and (2)). This leads to an exponential decay of viral load, determined by the half-life of actively infected cells, after a short shoulder phase [8,9] (Figure 1B). The shoulder phase is influenced by the clearance rate of free virus in the plasma [10], and can be affected by factors such as drug absorption into the plasma and diffusion into cells and targeting to lymphoid compartments and crossing the blood–brain barrier. Early studies, coupled with frequently sampled viral load data from patients on initial ART drugs, estimated the lifespan of infected cells to be around 2.5 days [8,52]. Subsequent studies with more potent drug combinations adjusted this estimate to approximately 1 day [53]. These findings highlighted the dynamic nature of HIV-1 infection, as each dying infected cell needs to be replaced by a newly infected one to maintain steady-state viral loads.

Overall, ART acts to block the production of new infected cells, effectively reducing β in the viral dynamics model (Figure 1, Equation (2)). Assuming fully effective treatment, with β→0, the basic viral dynamics model predicts [8,52] that viral load decays following

(3) $$V\left(t\right)=V\left(0\right)\frac{ce^{-d_{I}t}-d_{I}e^{-ct}}{c-d_{I}}$$

**Figure 2:**
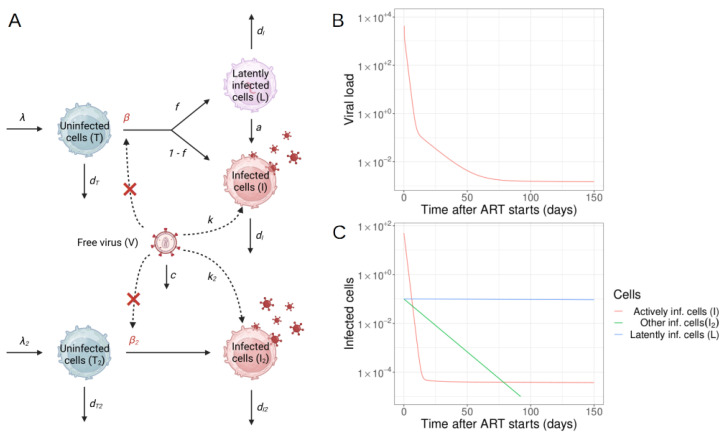
Schematic of a viral dynamics model involving multiple populations of infected cells. (**A**) A flow diagram between two populations of uninfected cells (T, T${}_{2}$), virus infected cells (I, I${}_{2}$), a latently infected cell population (L) and free HIV-1 (V) according to the ODE shown in Equation (4). Red X’es indicate the complete interruption of viral infection during fully effective therapy. (**B**) The decay of the viral load in multiple stages is shown. (**C**) The decay of distinct host-cell populations, as predicted by the model, are depicted. Time-series were integrated using JSim v2.21 with standard integration parameters [32]. (Figure adapted from Hill [18] and created by part (**A**) with BioRender.com).

While this equation describes the first phase (first week) of viral decay well (e.g., [53]), longer follow-up periods show that the decay slows over time. As more sensitive viral load assays were developed and drug combinations that prevented rapid resistance evolution were used, it was observed that viral load decline did not follow a simple exponential decay. Instead, a multi-phasic decay pattern was observed and best fit the data. Mathematical models were developed to explain this pattern, considering different populations of infected cells with varying death rates (Figure 2). A second phase decay, representing a population of virus-producing cells with a half-life of 14 days, was initially identified [11,54]. Based on this decay rate and estimated total body infected cell population size, it was speculated that complete viral eradication could occur within three years [11].

Perelson et al. [11], as well as other researchers, have developed more realistic models that incorporate multiple populations of infected cells to help explain these dynamics (Figure 2 and Equation (4)). A fraction (*f*) of new infections of target cells (i.e., CD4 T cells) result in latency. Latently infected cells (L, typically resting memory CD4 T cells) do not actively produce virus and are extremely long-lived. Occasionally (at rate *a*), these cells reactivate and produce virus. An alternative population of target cells, such as dendritic cells [55], hematopoietic stem cells, macrophages (although controversial) [56], turns over more slowly and produces virus at a lower rate. The resulting equations are

(4) $$\begin{array}{ccc} \dot{T} & = & \lambda -\beta TV-d_{T}T\\ {\dot{T}}_{2} & = & \lambda_{2}-\beta_{2}T_{2}V-d_{T_{2}}T_{2}\\ \dot{I} & = & \left(1-f\right)\beta TV-d_{I}I+aL\\ \dot{L} & = & f\beta TV-d_{L}L-aL\\ {\dot{I}}_{2} & = & \beta_{2}T_{2}V-d_{I_{2}}I_{2}\\ \dot{V} & = & kI+k_{2}I_{2}-cV \end{array}$$

The selected parameters of this model are λ=100 cells/µL, $\beta =10^{-7}$ or 0/ day/(virus/mL), k=1000 virus/cell, $d_{T}=0.1$/day, $d_{I}=1$/day, $\lambda_{2}=0.01$ cells/µL, $\beta_{2}=10^{-7}$ or 0/day/(virus/mL), $k_{2}=100$ virus/cell, $d_{T_{2}}=0.01$/day, $d_{I_{2}}=0.1$/day, c= 25/day, $f=10^{-4}$, $a=4\times 10^{-4}$/day, $d_{L}=10^{-4}$/day.

When fully effective therapy is given (β, $\beta_{2}\to 0$), viral load decays as

(5) $$V\left(t\right)=V\left(0\right)[Ae^{-d_{I}t}+Be^{-(a+d_{L})t}+Ce^{-d_{I_{2}}t}+\left(1-A-B-C\right)e^{-ct}]$$

where $T_{0}$ is the CD4 T cell level at the time of treatment start and

(6) $$\begin{array}{ccc} A & = & \frac{k\beta T_{0}}{\left(c-d_{I}\right)d_{I}}\left(1-\frac{(d_{I}-d_{L})f}{\left(d_{I}-d_{L}-a\right)}\right)\\ B & = & \frac{afk\beta T_{0}}{\left(a+d_{L}\right)\left(d_{I}-a-d_{L}\right)\left(c-a-d_{L}\right)}\\ C & = & \frac{c-\frac{k\beta T_{0}}{d_{I}}\left(1-\frac{d_{L}f}{\left(a+d_{L}\right)}\right)}{c-d_{I_{2}}} \end{array}$$

However, later studies using more sensitive assays identified a third phase of decay with a longer half-life (40–60 weeks) and potentially a subsequent stable viremia level [6, 88]. These findings, along with the discovery of latent infection in resting CD4 T cells [1,2,57] and viral rebound upon ART interruption [58,59], diminished hopes of a cure for HIV-1 via ART. The identity of the later phases of viral decay is still not fully understood. The second phase remains elusive, and it could represent longer-lived infected cell populations such as monocytes, macrophages, partially activated T cells, or cells with unintegrated HIV-1 DNA [15,60]. The third and fourth phase is likely due to reactivation of infection in long-lived latently infected cells (see also Section 2.2 below).

In summary, mathematical models have contributed to understanding the issue of multi-phase viral dynamics. For example, interpretations of decay kinetics changes when integrase inhibitors have been used instead of reverse transcriptase inhibitors to suggest that macrophages [61] and pre-integration latency [54] are unlikely causes for the second and third phase decay, respectively. Alternative hypotheses have been proposed based on mathematical modeling. These include the idea by Arnaout et al. [62] that decreasing antigenic stimulation of cytotoxic T cells leads to a decrease in viral load decay rate after ART initiation. Lower cytotoxic T cell levels would result in less killing of productively infected cells, and hence an apparent decrease in viral load decay rate. Kim and Perelson [14] hypothesized that viremia during the later phases was due to reactivation of infection from latently infected memory CD4 T cells specific to different antigens at different time points. The latent pool would then be comprised cells specific for rare antigens which appear infrequently, resulting in decelerating decay over time. Zhang and Perelson [63] suggested that the third phase of viral load decline could be explained by very slow release of virus bound to the surface of follicular dendritic cells due to complex multi-valent binding kinetics.

### 2.2. Mechanisms of Latent Reservoir Persistence

The persistence of residual viremia, even during effective combination ART [64], has been a significant focus of HIV-1 research for the past two decades. The understanding of HIV-1 latency has shed light on this phenomenon but has also raised new questions that remain unanswered to this day. Mathematical models have played a crucial role in investigating various hypotheses related to HIV-1 persistence. When ART is initiated, plasma viral loads typically decrease dramatically (by approximately 1000-fold) with a half-life of a few weeks. This initial drop is likely due to the suppression of actively replicating virus. However, even after about six months of treatment, a more stable population of infected cells is revealed, persisting at a frequency of approximately one in a million. This reservoir of latently infected cells decays extremely slowly, with a median half-life of about 44 months [3,57,65]. These findings have significant implications for HIV-1 treatment strategies. The slow decay of the latent reservoir indicates that the duration of therapy required to achieve a cure (which has been estimated to be around 70 years) is impractical for clinical purposes. Additionally, it has been observed that if ART is discontinued, viremia rapidly rebounds to pre-treatment levels [58,59], underscoring the need for lifelong ART. The discovery of this persistent viral reservoir has sparked ongoing research efforts to understand its sources and develop strategies to eliminate or control it. Despite significant advancements, many questions regarding the mechanisms of HIV-1 latency and reservoir persistence remain unanswered. Mathematical models continue to be valuable tools for testing various hypotheses and guiding future research in this field.

**Figure 3:**
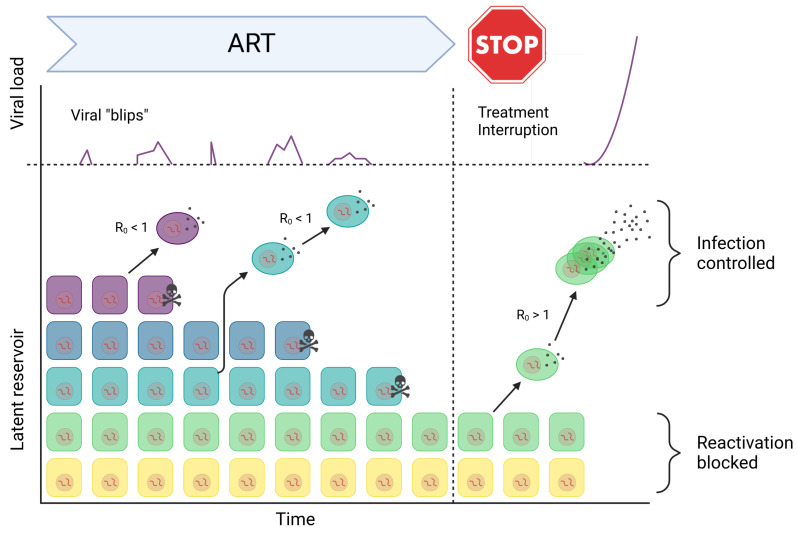
Schematic of latent reservoir dynamics. The latent reservoir involves long-lived resting memory CD4 cells, with potentially integrated HIV-1 provirus. At subcritical viral replication rate ($R_{0}<1$), the persistence of virus represents the maintenance of the latent reservoir. Infected host cells within this reservoir may occasionally die (marked by skull and bones), proliferate, or reactivate. A large proliferation rate leads to a decrease in viral diversity within the latent reservoir. New infections (bursts) are either completely blocked (Reactivation blocked) or may occasionally occur by stochastic processes. But continuous chains of replication are inhibited in the $R_{0}<1$ regime (Infection controlled). After treatment interruption ($R_{0}>1$), reactivated cells can produce virus that infects other host cells yielding to exponential growth in viral load (see Section 4, in particular Figure 4C). (Figure adapted from Hill [18] and created with BioRender.com).

Following the recognition of the persistence of latent HIV-1 and residual plasma viremia despite highly suppressive therapy, researchers have sought to understand the underlying mechanisms (Figure 3). Two main hypotheses have emerged in this regard. The first hypothesis suggests that latent infection is maintained by mechanisms similar to immunologic memory. According to this theory, the occasional reactivation of latent cells leads to the production of new virus particles, resulting in residual viremia. This hypothesis implies that the reservoir of latently infected cells serves as a long-term source of viral production. The second hypothesis proposes that ART is not completely effective in halting viral replication, indicated by an effective reproduction number ($R_{0}$) greater than 1 (see also Section 1 and Equation (2)). In this scenario, continual rounds of infection can lead to persistent viremia or persistent latent infection, or even both concurrently [66,67]. This hypothesis suggests that despite the suppression of viral replication by ART, ongoing low-level replication may contribute to the maintenance of viral reservoirs and residual viremia [68,69]. However, the topic of ongoing viral replication under ART is still up for debate, because careful analyses of viral evolution in patients on ART appear incompatible with ongoing replication during ART [70].

A substantial body of research utilizing mathematical modeling has focused on understanding the relationship between the dynamics of latently infected cells and plasma viremia during ART [13,14,17,71,72,73]. These modeling studies have provided valuable insights into the persistence of latent virus and its impact on plasma viral levels. One key finding from these studies is that when ART is marginally effective, the longevity of infected lymphocytes and the rate of their reactivation are the primary factors influencing HIV-1 persistence. The models suggest that the reservoir of latently infected cells is primarily maintained by the survival of these cells over time, rather than by ongoing viral replication continually seeding the reservoir. In other words, the presence of latently infected cells, rather than ongoing replication, is the major contributor to the persistence of the reservoir. The models further predict that low levels of ongoing viral replication ($0<R_{0}<1$) may influence the detectable levels of plasma virus. However, the models also suggest that these low levels of replication are highly unlikely to allow for the observed long-term sequence evolution of the virus, such as the development of drug resistance [13,14,72,73]. This finding emphasizes that the primary source of persistent viremia during ART is the reactivation of latently infected cells rather than ongoing replication. Clinical studies have observed fluctuations in residual plasma virus levels during ART, occasionally showing temporary increases known as viral “blips”. Mathematical models have been instrumental in explaining these observations, suggesting that these blips can be attributed to the occasional reactivation of latent cells driven by antigen stimulation. These models provided insights into the size, duration, and frequency of these blips, highlighting the role of antigen-driven reactivation of latent cells [13,14,17,71].

In the previous sections, we have described the contribution of cellular aspects to mathematical modeling including the dynamics of active infection and clearance of host cells as well as the contribution of latently infected cell populations to the decay kinetics. Below, we explain the molecular processes that contribute to cellular phenotypes in actively and latently infected cells.

## 3. Molecular Features Contributing to HIV-1 Latency and Reactivation

Molecular processes are essential components in the life cycle of the virus, from binding to target cell receptors, fusion of the viral and cellular membrane, release of the viral core, reverse transcription of the viral RNA, formation of a pre-integration complex, import into the nucleus, integration into the host genomic DNA, transcription, RNA export and translation, transport of proteins and genetic material into the cytoplasm for assembly, budding and maturation of the virus [74]. These processes can and have been addressed in a phenomenological matter by cellular models but the required details to fully understand the infection and to discover pathways for a possible cure are missing. For example, the development of novel drugs, in particular, to address the persistence of the latent reservoir, requires a molecular approach to identify and manipulate viral and/or cellular factors that regulate HIV-1 transcription [75].

Transcription is one critical regulatory process of the viral life cycle. Several regulatory features were previously identified to influence proviral transcription in cis and/or in trans, thereby contributing to HIV-1 transcription, latency maintenance, and/or reactivation. Below we describe them one-by-one and discuss what features have been incorporated into the various mathematical models, what have we learned and what remains to be learned.

### 3.1. Integration Site

The virus integrates in a semi-random manner in the genome of infected cells [76,77], mainly in chromatin accessible sites. Given the heterogeneity of the integration landscape, it has been seen as a major feature regulating proviral fate through position effects [78]. Pioneering work by Verdin and colleagues have provided evidence for this phenomenon [79]. They have used an HIV-1-derived retroviral vector, in which the GFP protein was under the control of the HIV-1 long terminal repeat (LTR) promoter, to generate multiple clonal cell lines each containing a single integrated virus. These clones showed large differences (up to 75-fold) in GFP expression levels between the highest and lowest expressing clones, both in the basal promoter activity and Tat inducibility, thus suggesting differences in the chromatin environment surrounding the provirus may regulate both basal and Tat functions. Various factors, such as transcription factors (TFs), RNA polymerase II (RNAPII) and chromatin (both locally in the provirus and more distally in its neighborhood), as well as nucleosome positioning and histone modifications, can perhaps determine the permissiveness of the chromatin environment for transcription control [80]. By simultaneously profiling the integration sites and transcriptional activity of individual proviruses at the single-cell level, Einkauf et al. described a global genomic and epigenetic map of transcriptionally active and silent proviral species in persons under ART [81]. As such, the integration site provides the coordinates for the assembly of transcription machinery and chromatin-based regulation, both critical features that modulate viral transcription.

### 3.2. Chromatin

Chromatin can be divided into proviral chromatin and the chromatin environment surrounding the provirus neighborhood both upstream and downstream. Proviral chromatin is composed of the nucleosomes that assemble on proviral DNA including the Nuc-0, which is located in the enhancer/modulatory regions, and Nuc-1, which is positioned right after the Transcription Start Site (TSS) and overlapping the TAR element [82,83] (reviewed in [84]). These nucleosomes and others formed with the proviral genetic material regulate viral transcription in cis. Further, the chromatin environment surrounding the provirus can influence the ability of the transcriptional machinery to access and initiate transcription in trans [85]. The position of genomic domains such as enhancers, silencers, and insulators together dictate the landscape of histone modifications at the chromatin environment, which assemble in the three-dimensional space as part of topological-associated domains (reviewed in [84]). Thus, both proviral chromatin and the surrounding chromatin environment are what probably determine the final composite of nucleosome positions dictating chromatin accessibility. Although not discussed in detail here, it is possible that this feature is also influenced by long-range chromatin interactions that arise as a consequence of communication between the provirus and/or surrounding neighborhood with other sites in the same and/or other chromosomes (trans effect). However, it remains unclear how the proviral chromatin is influenced in a context-dependent manner by the chromatin environment. Incorporation of these critical regulatory features in mathematical models will require advanced efforts.

Given that the DNA sequence dictates nucleosome positioning [86] and the known HIV-1 genetic variability [87], it is possible that proviral sequences also contribute to variable transcriptional responses either in a cell-type specific manner or more broadly. However, incorporation of this feature to mathematical models is currently impossible, as we need to first understand the basics of the process regulated by chromatin and transcription machinery.

### 3.3. Transcription Machinery

During latency in resting cells, the provirus is typically found in a repressed chromatin state, which restricts Tat synthesis. HIV-1 exit from latency is characterized by a rapid transition in cell signaling and transcriptional activity, whereby master TFs acutely translocate from the cytoplasm into the nuclei (e.g., NF-kB and NFAT) and/or are activated through gene expression and post-translational modifications (e.g., AP-1). Binding of these TFs to their cognate cis-elements at the provirus facilitates several steps in the transcription activation cycle leading to latency reactivation including the assembly of the transcription pre-initiation complex (PIC) with general transcription factors (GTFs). RNAPII is recruited to the proviral genome through interactions with select GTFs including TBP/TFIID, TFIIA and TFIIB, and then escapes the promoter by losing contacts with the PIC before it pauses a few nucleotides downstream the TSS; [88,89], however, the pause site has not yet been characterized at high resolution. RNAPII pausing is not a stable state, and RNAPII molecules that do not commit intro productive elongation in the absence of Tat undergo premature termination [90]. RNAPII pausing is transiently enforced by the negative elongation factor (NELF) and DRB sensitivity-inducing factor (DSIF) (reviewed in [91]). In the presence of Tat in reactivated cells, P-TEFb is recruited to the nascent TAR RNA where the kinase phosphorylates NELF, DSIF, and RNAPII, thereby triggering pausing exit and processive elongation through concerted association with the Super Elongation Complex (SEC) [92,93,94,95,96].

Together, our understanding of the molecular features regulating HIV-1 proviral transcription have provided important clues for devising mathematical models as described in the section below.

## 4. Transcriptional Bursting and Mathematical Modeling

### 4.1. Transcriptional Bursting and Gene Expression Noise

The above section briefly described the molecular features that could regulate the process of HIV-1 transcription. In this section, we focus on the modes of HIV-1 transcription during latency and reactivation. Transcriptional bursting, which refers to the intermittent production of bursts of transcripts [97], is a characteristic feature of HIV-1 transcription [92,98], and is a major source of gene expression heterogeneity [99,100,101]. Transcriptional bursting appear to be regulated by a plethora of molecular factors, including TF regulation [102,103], chromatin environment [101], nucleosome positioning [104,105], and regulation of promoter-proximal RNAPII pausing [106,107] and recycling [108].

The chromatin environment can modulate the frequency and magnitude of transcriptional bursting by influencing TF binding, nucleosome positioning, and RNAPII recruitment and pausing [109,110]. In latent cells, it has been proposed that the chromatin environment is repressive with lower accessibility [89], and that RNAPII promoter-proximal pausing prevents proviral transcription and is essential for latency maintenance [92]. In reactivated cells, the chromatin environment switches to an active conformation and accessibility increases, and the viral factor Tat releases paused RNAPII to induce proviral transcription. The behavior of RNAPII pausing at latent proviruses was proposed to be stochastic (stochastic pausing), thus generating transcriptional bursting, which then contributes to the stochastic reactivation of latent HIV-1 [92].

Host TFs play crucial roles in modulating transcriptional noise by recruiting molecular complexes that affect various aspects of transcriptional bursting. Several studies have explored how TFs influence inducible gene expression noise, but they often lack a direct connection between molecular details in the transcription process. When a signal from the immune microenvironment (e.g., the pro-inflammatory cytokine TNFα) initiates a cell signaling cascade, it activates the master TF NF-κB, which operates through a multi-layer regulation of essential steps to activate the provirus, including binding to the LTR, recruiting histone acetyltransferases (HATs) such as CBP/p300, and recruiting the elongation factor P-TEFb, among others (reviewed in [111]). The recruitment of HATs leads to the destabilization of DNA-histone interactions within nucleosomes, increasing the accessibility of the promoter region. Once the promoter becomes accessible, NF-κB then facilitates the recruitment of the Mediator complex [112], RNAPII, and other components of the PIC for assembly of the transcription machinery at the LTR. Recruitment of P-TEFb then releases paused RNAPII and enables efficient transcriptional elongation to proceed (reviewed in [91]).

The mechanism of transcriptional bursting produces gene expression noise. The effect of endogenous noise in cellular processes in general, and gene expression heterogeneity in particular, was predicted as early as the 1970s by Spudich and Koshland [113]. The theoretical analysis [114] and the direct measurement [115,116] of the physical basis of noise sources required 20 more years. Studies on the λ-phage, a bacterial virus, have provided a paradigmatic example of how stochastic thermal fluctuations in chemical reaction rates can influence the life-cycle decision between lysis and lysogeny. The life cycle of the λ-phage involves two distinct paths: the lytic cycle, where the virus replicates within the host bacterium and eventually lyses it, releasing new viral particles, and the lysogenic cycle, where the phage DNA integrates into the host genome and remains dormant. Stochastic expression from phage λ’s divergent pR and pRM promoters is critical in controlling the λ switch, and can explain the phage’s non-deterministic lysis/lysogeny choice [117]. Subsequent experimental measurements of expression from the λ promoter and its operation in synthetic circuits confirmed its fundamentally stochastic nature [118].

In summary, transcriptional bursting and gene expression noise are intrinsic features of the HIV-1 transcriptional program and are regulated by a myriad of molecular processes contributing to the stochastic nature of HIV-1 latency reactivation.

### 4.2. A Stochastic Model to Describe the HIV-1 Transcriptional Circuit

Weinberger et al. explored whether HIV-1 latency can be explained by stochastic gene expression [119]. For this purpose, they used an HIV-1 model vector (LTR-GFP-IRES-Tat-LTR) and employed a stochastic system based on the Gillespie algorithm [120]. Their stochastic mathematical model described dynamics of the nuclear and cytoplasmic HIV-1 mRNA concentrations of a reporter protein driven off the HIV-1 LTR in the presence of Tat as well as the reporter GFP protein. The corresponding ODE, including the positive Tat feedback loop, is shown below [119]

(7) ddt[Tat]=−dtt·[Tat]ddt[nRNA]=b+v·[Tat]k+[Tat]−ex·[nRNA]−dr·[nRNA]ddt[cRNA]=ex·[nRNA]−dr·[cRNA]ddt[P]=vp·[cRNA]kp+[cRNA]−dp·[P].[RNA]=[nRNA]+[cRNA]

with dtt=0.154; b=0.01; dr=1.6; ex=2.6; dp=0.39; v=150; k=50; vp=11; kp = 0.676.

Weinberger et al. found that low levels of viral gene expression (measured by GFP reporter protein encoded by the provirus), resulted in two distinct phenotypes within clonal populations derived from single viral integrations. Some cells exhibited very high GFP expression, while others showed near-zero GFP expression. This phenomenon, termed phenotypic bifurcation (PheB), was observed even though the cells had the same proviral integration patterns and did not apparently differ in cell-intrinsic factors such as cell cycle, cell size, aneuploidy, or chromatin silencing. The stochastic modeling approach successfully accounted for the observed PheB and accurately predicted the dynamics of a Tat mutant. The predictions made by the model were later confirmed through experimental validation. These findings suggest that stochastic fluctuations in Tat expression alone are sufficient to generate PheB and potentially contribute to the establishment of latency. Weinberger et al. highlighted the significance of stochastic gene expression fluctuations in a mammalian system, emphasizing the importance of considering such variability when studying HIV-1 gene regulation [119].

### 4.3. Incorporating Host and Viral Phases to Model the HIV-1 Transcriptional Circuit

**Figure 4:**
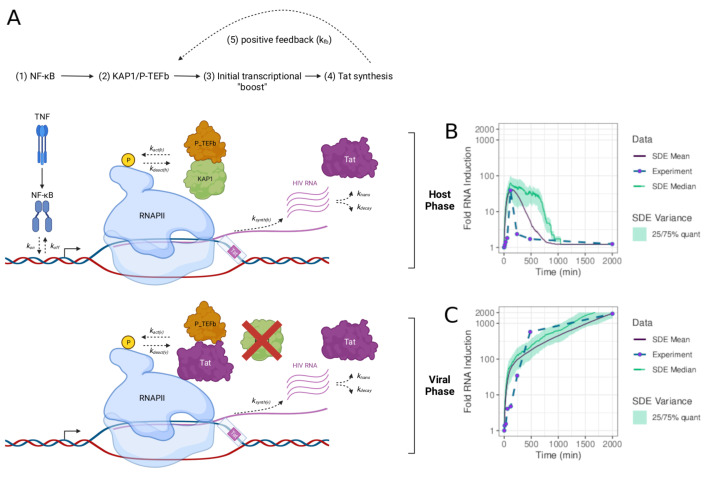
Mathematical model of the host and viral phases of the HIV-1 transcriptional program [121]. (**A**) Simplified model of the host and viral phases of the HIV-1 transcriptional program. (**B**) Experimental data and fitted stochastic computer simulation of a host cell infected by HIV-1 in the host phase without feedback by Tat. (**C**) Experimental data and fitted stochastic computer simulation of a host cell infected by HIV-1 in the viral phase with feedback by Tat.

While Weinberger et al. focused on the two states (basal and Tat-activated), Morton et al. were further interested in a more comprehensive understanding of the HIV-1 transcriptional circuit [121]. For this purpose, they expanded the simpler Weinberger et al. model describing the “basal-viral” phases, which takes place during normal cell homeostasis that maintains a low level of non-productive RNA synthesis by “basal” steady-state transcription [119]. Gene expression in the basal phase leads to short, immature transcripts (Figure 4). In this phase, the viral activator Tat is not expressed and, thus, HIV-1 does not replicate (latent state). A second phase is the “host” phase. There, in cells exposed to immune stimulation, master TFs like NF-κB and NFAT are activated, leading to an initial low-level “boost” in proviral transcription. In proviruses lacking Tat, this phase shows a unimodal pattern of activation that is quickly turned off, leading to a small number of viral products (Figure 4).

During productive infections with wild-type proviruses, the initial transcriptional boost is critical because it enables Tat synthesis before the host phase turns off. In this case, the host phase is rapidly followed by a “viral” phase in which Tat amplifies transcription by more than 100-fold, promoting a positive transcriptional feedback loop and robust viral replication (Figure 4). Considering all three phases of HIV-1 transcription, Morton et al. investigated the roles of host cell factors, the immune cell stimulation on the host phase and its effect on the positive feedback loop [121]. In particular, Morton et al. focused on the dynamics of the non-basal expression depending on activation by NF-κB, the host transcriptional regulator KAP1 (TRIM28, TIF1β) and Tat

(8) d[RNA]dt=μRNA[NFκB]kMm+[NFκB]+τRNA[KAP1][NFκB]+ksynth(h)[KAP1][Tat]+ksynth(v)[Tat]−kdecay[RNA]d[Tat]dt=ktrans[RNA]+μTat[Tat]kMTat+[Tat]−dTat[Tat]d[NFκB]dt=β[TNF]−dNFκB[NFκB]d[KAP1]dt=ρ−dKAP1[KAP1]d[TNF]dt=−dTNF[TNF]d[RNAbasal]dt=α−dRNAbasal[RNAbasal].[RNAtot]=[RNA]+[RNAbasal]

Parameters are listed in Table S1.

They found that variable levels of KAP1 in several clonal cell lines influenced the magnitude and homogeneity of HIV-1 latency reversal potential (referred to as transcriptional fragility). The findings by Morton et al. [121] illuminated that the host phase of the HIV-1 transcriptional program plays a significant role in ensuring that the virus completes its pathogenic cycle. Thus, its understanding is crucial for studies of HIV-1 latency and efforts for a functional cure.

### 4.4. Two-State HIV-1 Transcriptional Model

Several mathematical models have been devised to attempt to define and understand the complexity of the HIV-1 transcriptional program. In the context of inducible gene expression, a two-state promoter model (on–off) has been initially created. This model is limited in its ability to capture the complexity of transcriptional regulation and thus falls short in capturing the intricate molecular mechanisms involved in TF-mediated inducible HIV-1 gene expression and reactivation from latency. This model does not directly account for the recruitment of specific molecular complexes by TFs or the subsequent effects on transcriptional bursting dynamics, including burst size and burst frequency. To capture these details, more sophisticated models are needed, integrating the precise temporal steps including TF activation, recruitment of co-activators and RNAPII, chromatin-based regulation, and transcriptional elongation, among others. These complex models may help thoroughly elucidate the precise regulatory mechanisms underlying latent HIV-1 transcription and provide a more accurate representation of transcriptional noise.

### 4.5. Three-State HIV-1 Transcriptional Model

**Figure 5:**
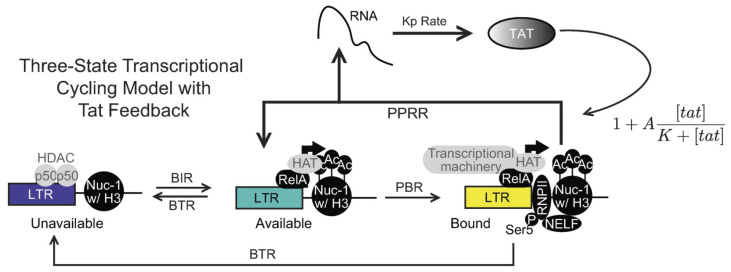
Positive feedback on the RNAPII pause release rate (PPRR) activation does not influence bimodality of the mRNA and protein distributions in the three-state transcriptional cycling model [109]. Updated three-state promoter system with HIV-1 nucleosome remodeling, RelA recruitment, and Tat-mediated transcript elongation, which is amplified via positive feedback. Positive feedback is modeled as a saturating function with an amplitude, *A*, and half-max, *K*.

Bullock et al. [109] explicitly considered the effect of chromatin accessibility and RNAPII pausing on transcriptional noise, exploring a previously published three-state promoter model [107] (Figure 5). This model describes transcription regulation by a promoter which can alternate in three states: (1) the unavailable promoter (UP), (2) the available promoter (AP), and (3) the bound promoter (BP). The transitions between these states determine the promoter activity. The rate at which the UP state transitions to the AP state is referred to as the burst initiation rate (BIR), while the rate at which the AP state transitions back to the UP state is the burst termination rate (BTR). These rates reflect the remodeling of the chromatin environment surrounding the promoter, which includes processes like nucleosome repositioning and chromatin modifications (e.g., histone deacetylation). When the AP state is occupied by RNAPII and the associated transcriptional machinery, it transitions to the BP state at the RNAPII binding rate (PBR). The BP state represents an initiated but paused promoter, where transcription is temporarily halted. The rate at which the promoter releases the paused RNAPII and returns to the AP state is the RNAPII pause release rate (PPRR). Once in the BP state, elongated transcripts are produced and translated into proteins at a rate denoted as Kp. The model considers that only one RNAPII molecule can bind to the promoter at a time, and it remains paused until it is released, and that the degradation of both mRNA and proteins, assuming a first-order process.

The model describes two possible scenarios [107]. In the first scenario, the promoter continuously cycles between the AP and BP states, resulting in bursts of transcription. Hence, the model is referred to as a transcriptional cycling model. In the second scenario, the BP state transitions back to the UP state, indicating a burst termination event. The BTR for this transition is assumed to be the same as the BTR for the AP state to UP state transition, as the biological processes governing these transitions, such as TF removal and chromatin remodeling, are similar to each other and distinct from the processes governing the other transition rates. Bullock et al. [109] expanded the original model by Bartman et al. [107] and included a Tat-mediated positive feedback to the transcriptional cycling model by amplifying *PPRR* with a Tat-dependent term (Figure 5) as shown

(9) $$Feedback=PPRR\times \left(1+A\frac{\left[TAT\right]}{K+[TAT]}\right)$$

In conclusion, mathematical models have allowed us to decipher the interplay of multiple features contributing to transcriptional bursting and stochasticity, the multiple promoter states and the switch between different phases of the transcriptional cycle. These features have helped elucidate, at least in part, the stochastic nature of HIV-1 latency and reactivation. These methods will further aid in the development of novel therapeutic strategies, such as the use of noise suppressors for “block and lock” cures [122].

## 5. Discussion and Future Perspective

Elucidating the molecular processes that shape latency establishment, maintenance and reactivation is essential in our race towards a sterilizing cure. Mathematical modeling has played an instrumental role in our understanding of HIV-1 infection, pathogenesis, persistence and latency dynamics, including the roles of the HIV-1 transcriptional program during latency maintenance and reactivation. Transcriptional bursting is a bona fide feature of the HIV-1 transcriptional program [92,98] and an evolutionary conserved transcriptional control feature (reviewed in [97]).

The occurrence of transcriptional bursts during periods of latency and reactivation are affected by stochastic molecular processes. One current model in the field is that the stochastic activation of HIV-1 in latently infected cells can re-establish viral propagation, thus preventing patients from clearing the virus. The generation of transcriptional bursts by stochastic activation of the viral promoter is responsible, in part, for the heterogenous latency exit [119,123]. Thus, understanding the interplay between TF assembly, chromatin environment, transcriptional bursting, and HIV-1 latency maintenance and reactivation is crucial for developing strategies to target and eliminate the latent reservoir.

Several features appear to regulate transcriptional bursting during latent HIV-1 re-activation including genomic elements (enhancer and promoter) and their structures, the transcriptional machinery that assembles at those sites, and the chromatin microenvironment surrounding integrated proviruses (both nucleosome positioning dictating chromatin accessibility at, and the collection of histone modifications surrounding, the 5${}^{\prime }$-LTR). These features may differentially influence the size and frequency of bursting events, which are key parameters of the two-state and three-state models of viral transcription. However, advancement of modeling and analysis tools has revealed that the simple two-state model and associated parameters may not sufficiently recapitulate the complex relationship between these features. As such, more complex models incorporating some of these features such as RNAPII pausing and chromatin have been devised (reviewed in [124]). A common outcome of all these complex models is the realization that the HIV-1 transcriptional program is fragile and that perturbations at any of its regulatory phases: basal, host, and/or viral (Figure 4) alters the magnitude of latent HIV-1 exit from latency, likely contributing to spontaneous reactivation (viral blips) [80,92,98,106,109,121,125,126,127].

A particular aspect of these multi-state models are their implication for potential therapeutic strategies. Razooky et al. [128] discussed therapeutic implication’s based on two-state k${}_{ON}$–k${}_{OFF}$ models. Tat based strategies altering k${}_{OFF}$ together with conventional latency-reversing agents would be optimal for “shock-and-kill” strategies, whereas conversely increasing k${}_{OFF}$ and decreasing k${}_{ON}$ would be optimal for “block-and-lock” strategies. Cao et al. [129] further suggested to probabilistically control HIV-1 latency and reactivation by different perturbations of the underlying probability landscape. They suggested effective therapeutic targets for strategies of “shock-and-kill” to eliminate latently infected cells and “block-and-lock” to enforce deep latency.

An outstanding question of several landmarks discoveries in the field, including the topic of stochasticity [124], is what selective pressures shaped the viral genome to be regulated in such a manner and what are the evolutionary advantages. This is extremely important, given that two distinct patterns of gene activation (synchronous and stochastic) have been proposed to regulate gene expression in Drosophila [130] and mammals (reviewed in [97]). Interestingly, while synchronous genes display essentially uniform expression of nascent transcripts in all cells of a Drosophila embryonic tissue, stochastic genes display erratic patterns of de novo activation. RNAPII is “pre-loaded” (stalled) in the promoter regions of synchronous genes, but not stochastic genes. This discovery argues against the model of RNAPII stable pausing at the proviral genome [92], and may suggest revisiting the contribution of RNAPII pausing to the importance of stochastic vs. synchronous HIV-1 transcriptional activation. While the concept of RNAPII pausing has been proposed to regulate entry into HIV-1 latency, pausing alone cannot explain the multi-layer complex regulation of transcription. Since RNAPII pausing blocks new initiation [131,132], there has to be a precise coordination between initiation, pausing, elongation and termination. Additional features such as regulation of elongation rate, termination, and reinitiation kinetics, will have to be incorporated to create a more accurate representation of the multi-level regulation of HIV-1 transcription and its importance to latency. Thus, future experimental evidence will be required to settle on a model, which will inform future mathematical modeling efforts to keep expanding our knowledge through synergistic experimental and modeling efforts. The activity of the multitude of regulatory features is intertwined with some dependence, thus models that include these considerations will outperform more minimalistic prototypes.

## Data Availability

All data are available at the cited sources.

## Supplementary Materials

The following supporting information can be downloaded at: https://www.mdpi.com/article/10.3390/v15102119/s1, Table S1: Parameters of the Morton et al. model [121].
